# Pals1 prevents Rac1-dependent colorectal cancer cell metastasis by inhibiting Arf6

**DOI:** 10.1186/s12943-021-01354-2

**Published:** 2021-05-04

**Authors:** Simona Mareike Lüttgenau, Christin Emming, Thomas Wagner, Julia Harms, Justine Guske, Katrin Weber, Ute Neugebauer, Rita Schröter, Olga Panichkina, Zoltán Pethő, Florian Weber, Albrecht Schwab, Anja Kathrin Wege, Pavel Nedvetsky, Michael P. Krahn

**Affiliations:** 1 Department of Medical Cell Biology, Medical Clinic D, University Hospital of Münster, Albert-Schweitzer-Campus 1-A14, 48149 Münster, Germany; 2 Institute of Physiology, University of Münster, Münster, Germany; 3 Institute for Pathology, University of Regensburg, Regensburg, Germany; 4 Department of Gynaecology and Obstetrics, University Medical Centre Regensburg, Regensburg, Germany

**Keywords:** Colorectal cancer, Metastasis, Cell migration, Pals1, Arf6, Rac1, Cytoskeleton

## Abstract

Loss of apical-basal polarity and downregulation of cell-cell contacts is a critical step during the pathogenesis of cancer. Both processes are regulated by the scaffolding protein Pals1, however, it is unclear whether the expression of Pals1 is affected in cancer cells and whether Pals1 is implicated in the pathogenesis of the disease.

Using mRNA expression data and immunostainings of cancer specimen, we show that Pals1 is frequently downregulated in colorectal cancer, correlating with poorer survival of patients. We further found that Pals1 prevents cancer cell metastasis by controlling Rac1-dependent cell migration through inhibition of Arf6, which is independent of the canonical binding partners of Pals1. Loss of Pals1 in colorectal cancer cells results in increased Arf6 and Rac1 activity, enhanced cell migration and invasion in vitro and increased metastasis of transplanted tumor cells in mice. Thus, our data reveal a new function of Pals1 as a key inhibitor of cell migration and metastasis of colorectal cancer cells. Notably, this new function is independent of the known role of Pals1 in tight junction formation and apical-basal polarity.

**Supplementary Information:**

The online version contains supplementary material available at 10.1186/s12943-021-01354-2.

## Results and discussions

Invasive cancer cells forming metastases in distant organs are the cause of most tumor-related deaths. Downregulation of cell-cell and cell-matrix adhesion contacts as well as loss of apical-basal polarity are hallmarks of metastasis. Consequently, expression of E-Cadherin (E-Cad) negatively correlates with patient survival and is used as a prognostic marker.

The adaptor protein Pals1 (Protein associated with Lin-7 One, encoded by *Membrane Protein Palmitoylated 5*, *MPP5*) stabilizes the transmembrane protein Crb (Crb3 in most classical epithelia) and links it to the Myosin-regulator PATJ (Pals1-associated TJ protein). Loss of the Crb complex results in disturbed or delayed formation of tight junctions (TJ) in cultured cells [1–4]. In contrast to Crb3, which has been linked to EMT [5–7], nothing is known about the implication of Pals1 in the pathogenesis of cancer.

### Pals1 is frequently downregulated in colorectal cancer

**Fig. 1:**
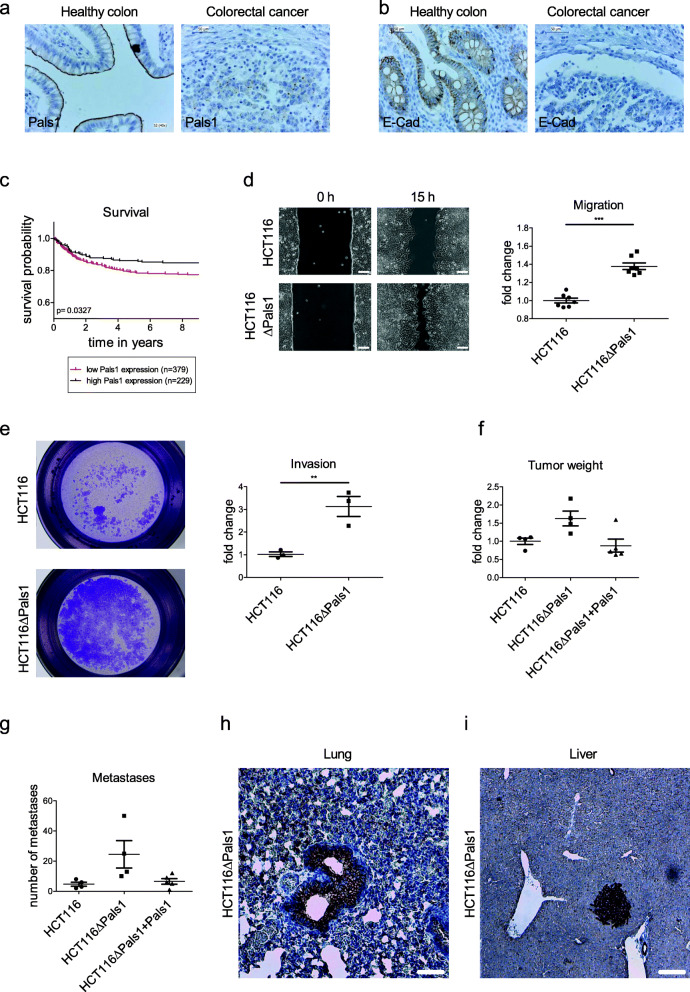
Pals1 inhibits cell migration and metastasis of colorectal cancer cells. **a** and **b** Representative IHC stainings of Pals1 and E-Cad in healthy colon tissue compared to colorectal cancer specimen. **c** Survival probability of colorectal cancer patients with high (5,7 FPKM (Fragments Per Kilobase of sequence per Million mapped reads) on average) or low (2,9 FPKM) Pals1 expression. Healthy control samples display on average 7,07 FPKM (not shown). **d** Representative images of a wound healing assay of HCT116 and HCT116ΔPals1 cell lines and the corresponding quantification (*N* = 7). **e** Representative images and quantification of a transwell matrigel invasion assay of HCT116 and HCT116ΔPals1 cell lines (*N* = 3). **f** Tumor weight and **g** average number of metastases found in liver and lung after subcutaneous transplantation of HCT116 and HCT116ΔPals1 in immunodeficient mice after 5 weeks (*N* = 4 for HCT116wt and HCT116∆Pals1 cells and *N* = 5 for HCT116∆Pals1 + Pals1 Rescue). **h** and **i** Representative pan-Cytokeratin IHC stainings of HCT116ΔPals1 cells colonizing in lung and liver. Scale bars are 20 μm

In order to elucidate the role of Pals1 in the pathogenesis of cancer, we first set out to examine its protein expression in poorly differentiated (G3) colorectal carcinoma specimen in comparison to healthy controls. Indeed, we found Pals1 expression to be decreased in the majority of evaluated samples (58% strongly reduced, *n* = 48, Fig. 1a), whereas in healthy enterocytes, Pals1 is strongly expressed and localizes at the apical membrane of enterocytes with an accumulation at apical cell junctions/TJ (100%, *n* = 11, Fig. 1a). E-Cad stainings show a similar reduction in highly dedifferentiated tumor samples compared to Pals1 (Fig. 1b). Notably, downregulation of Pals1 mRNA in a large colorectal cancer patient cohort (TCGA network [8]) correlates with a decreased survival compared to patients with high expression of Pals1 (Fig. 1c). Within this cohort (*n* = 483), only 6 missense mutations and one frameshift mutation leading to a truncation of *Pals1,* were identified, indicating that in colorectal cancer Pals1 is frequently downregulated but somatic mutations/truncations of Pals1 are rather rare.

### Deletion of Pals1 results in enhanced cell motility and migration in vitro and in vivo

In order to investigate the consequences of loss of Pals1 expression, we established a colorectal cancer cell line (HCT116) with Pals1 knockout using CRISPR/Cas9 (Supplementary Fig. 1a). The HCT116 cell line is derived from a Duke’s D primary tumor of colorectal origin but still expresses Pals1 (Supplementary Fig. 1a) comparable to tumor samples with high Pals1 expression (6,0 FPKM for HCT116 in comparison to 5,7 FPKM for colorectal cancer specimen with high Pals1 expression in Fig. 1c). In HCT116 cells, Pals1 is only partly accumulating at cell-cell contacts (Supplementary Fig. 1b-c). The TJ-adaptor protein ZO-1 appears fragmented, too, whereas the TJ transmembrane protein Claudin-7 is robustly localized to cell-cell contacts (Supplementary Fig. 1c). Notably, deletion of Pals1 does neither affect the localization of TJ proteins nor E-Cad expression and its accumulation at cell-cell junctions but the Pals1-binding partner PATJ is totally lost from cell-cell contacts (Supplementary Fig. 1b-e). Thus, deletion of Pals1 has only little impact on the residual apical-basal polarity and TJ assembly in this colorectal cancer cell line.

However, Pals1-deficient cells are more motile: Live imaging and tracking of individual cells reveal a more than twofold increased velocity and translocation (Supplementary Movie 1 and 2 and Supplementary Fig. 1f-h). Similar results were obtained in a two-dimensional scratch/wound healing assay (Fig. 1d). Moreover, Pals1-deficient cells display a threefold enhanced invasion potential when seeded on extracellular matrix (Fig. 1e). These data demonstrate an increased migration and invasion in vitro. In order to address, whether this in vitro capacity results in an enhanced metastasis of tumor cells in vivo, we implanted wild type HCT116 and HCT116∆Pals1 cells subcutaneously into immune-deficient mice and monitored tumor growth and the number of metastases found in lung and liver. The primary tumors derived from Pals1-deficient cells grew slightly bigger than these of control cells or HCT116∆Pals1 cells with Pals1 rescue transgene (Fig. 1f), which is in line with in vitro proliferation assays showing a higher plateau of HCT116∆Pals1 cells whereas the proliferation speed seems to be unaffected (Supplementary Fig. 1i). In contrast, differences in apoptosis obviously do not play a role in growth differences of Pals1 expressing and -depleted cell lines (Supplementary Fig. 1j). Remarkably, only HCT116∆Pals1 cells were capable of colonizing the liver, whereas no HCT116wt cells were found in this organ. The total number of metastases (in liver and lung) were dramatically increased for HCT116∆Pals1 cells (Fig. 1g-i), confirming a crucial role of Pals1 for metastasis of tumor cells in vivo.

### Pals1-deficient cells display an increased activation of Rac1

**Fig. 2:**
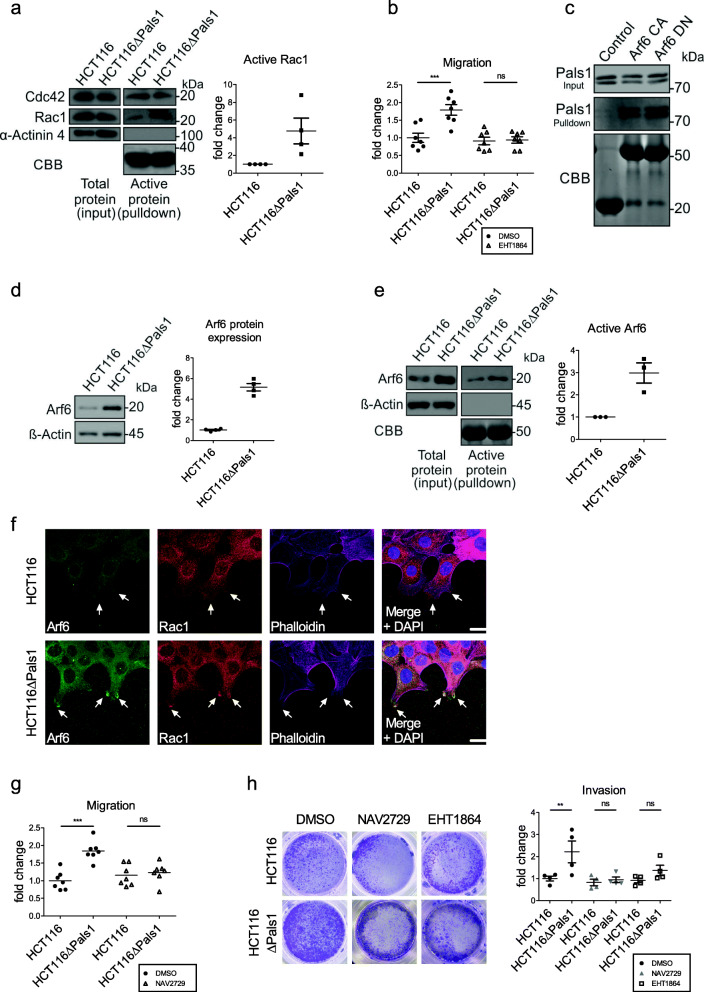
Pals1 inhibits Arf6 to control Rac1-dependent cell migration and invasion. **a** Western blot and quantification of pulldown assays for active Cdc42 and Rac1 using the protein binding domain (PBD) of p21 activated kinase 1 protein (PAK1) (*N* = 4). GST-PBD was stained with Coomassie brilliant blue (CBB). Active Rac1 was quantified by normalizing against α-Actinin 4. **b** Quantification of wound healing assays of HCT116 and HCT116ΔPals1 with the Rac1 inhibitor EHT1864 (5 μM) and DMSO as control (*N* = 7). **c** Western blot and CBB stained gel of a pulldown experiment using recombinant constitutively active (Q67L, CA) and dominant negative (T44N, DN) Arf6 fused to GST and wild type HCT116 cell lysates. GST alone served as control. **d** Western blot and quantification of Arf6 expression in HCT116 and HCT116ΔPals1 cell lines (*N* = 4). **e** Western blot and quantification of the pulldown assay for active Arf6 using the effector protein Golgi-localized γ-adaptin ear-containing, Arf-binding protein 3 (GGA3) (*N* = 3). GST-GGA3 is visualized by CBB staining. Active Arf6 was normalized against β—Actin. **f** Immunostainings of migrating HCT116 and HCT116ΔPals1 cells for Arf6 (green), Rac1 (red) and Phalloidin (magenta). Arrows indicate the tips of lamellipodia. **g** Quantification of wound healing assays with HCT116 and HCT116ΔPals1 incubated with the Arf6 inhibitor NAV2729 (10 μM) or DMSO as control (*N* = 7). **h** Representative images and quantification of transwell matrigel invasion assays of HCT116 and HCT116ΔPals1 cells with the Arf6 inhibitor NAV2729 (10 μM), the Rac1 inhibitor EHT1864 (5 μM) or with DMSO as control (*N* = 4). Scale bars are 20 μm

Cell migration and invasion is driven by the formation, retraction and re-formation of lamellipodia and the turnover of focal adhesions in these cell protrusions. Lamellipodia dynamics are regulated by Rac1-controlled Actin polymerization and -branching. Rac1 or its activating GEFs are upregulated or mutated in several types of cancer, including colorectal cancer, correlating with a poor prognosis for the patients [9]. Thus, we tested whether Pals1 deletion affects Rac1 expression or activation. Indeed, pull down experiments with the purified Cdc42/Rac1 interactive binding (CRIB) domain of p21 activated kinase (PAK), which selectively binds to GTP-bound, active Rac1 and Cdc42, revealed a fourfold increased activation of Rac1 in HCT116∆Pals1 cells while the activity of Cdc42 was unchanged (Fig. 2a and Supplementary Fig. 2a). In line with these results, the Rac1-dependent autophosphorylation of PAK is strongly increased in Pals1-deficient cells (Supplementary Fig. 2b). Finally, inhibition of Rac1 with EHT1864, which blocks Rac1 in its inactive state by direct binding, abolishes the increased migration (Fig. 2b) and invasion (Fig. 2h) of HCT116∆Pals1 cells, supporting our hypothesis that an ectopic activation of Rac1 in Pals1-deficient cells is the cause for the enhanced motility of these cells.

### Pals1 inhibits Arf6 in order to control Rac1-dependent cell migration

Apart from specific GEFs, Rac1 can also be activated by the small GTPase Arf6 [10]. Similar to Rac1, Arf6 is frequently overexpressed in several types of cancer, including invasive breast cancer, melanoma and pancreatic cancer and Arf6 activity is driving these tumor cells to become more invasive [11]. Pals1 has been found in a proteomic approach as an interaction partner of Arf6 [12], although the physiological relevance of this interaction has not yet been elucidated. We confirmed that recombinant Arf6 co-precipitates with endogenous Pals1 from HCT116 cells (Fig. 2c). Notably, the dominant negative, GDP-bound Arf6 variant co-precipitated similar amount of Pals1 as the constitutively active, GTP-bound form, indicating that binding of Pals1 is independent of the activation status of Arf6 (Fig. 2c). Depletion of Pals1 resulted in a fivefold increased Arf6 protein expression (Fig. 2d) and an increase in Arf6 mRNA by 2.5 (Supplementary Fig. 2c).

Furthermore, increased Arf6 protein levels correlate with increased total Arf6 activity in HCT116∆Pals1 cells, although the ratio of active Arf6/total Arf6 is not changed (Fig. 2e and Supplementary Fig. 2d). By contrast, total protein levels and the amount of active Arf1 are not affected (Supplementary Fig. 2f). Immunostainings of migrating wild type HCT116 cells reveal a faint colocalization of endogenous Pals1 and Arf6 at the plasma membrane of lamellipodia (Supplementary Fig. 2e, arrows) but not at cell-cell contacts, where only Pals1 accumulates (Supplementary Fig. 2e, arrowhead). Pals1-deficient cells exhibit an enhanced overall staining of Arf6 and a strong enrichment of Arf6 and Rac1 at the tips of lamellipodia (Fig. 2f, arrows). This is in line with the observation that overexpression of constitutively active Arf6 is sufficient to enhance lamellipodia formation and cell migration [11].

Finally, we tested whether increased Arf6 activity is the cause of the enhanced motility of Pals1-depleted cells. Indeed, incubation of HCT116∆Pals1 cells with the Arf6-specific inhibitor NAV2729 abolishes the differences in migration and invasion compared to the parenteral cell line (Fig. 2g and h) similar to incubation with the Rac1-inhibitor (Fig. 2b and h). In addition, downregulation of Arf6 in HCT116∆Pals1 cells significantly decreased migratory capacity of these cells like the Arf6 inhibitor (Supplementary Fig. 2 g-h). Taken together, these results suggest that Pals1 suppresses Arf6 in order to inhibit cell migration and invasion by controlling Rac1 activity.

### Arf6-mediated control of Rac1 activity by Pals1 seems to be a general mechanism

In order to address whether the increased activation of Arf6 and Rac1 is specific for HCT116 cells or whether it is a general mechanism important for tumorigenesis and metastasis of colorectal cancer cells, we generated another colorectal cancer cell line, SW48, with a knockout of Pals1 (Supplementary Fig. 3a). Indeed, Pals1-deficient SW48 cells display a similar increase in cell migration as HCT116 cells (Supplementary Fig. 3b). Furthermore, active Rac1 as well as Arf6 levels are strongly increased in SW48∆Pals1 cells (Supplementary Fig. 3c-d). However, in contrast to the situation in HCT116 cells, depletion of Pals1 in SW48 results only in a slight increase in total Arf6 expression (Supplementary Fig. 3e). In HCT116 cells, it is still unclear, how deletion of Pals1 results in an increase in Arf6 transcription. Enhanced Arf6 gene expression has been reported to be induced by activation of PI3K/Akt and ERK1/2 signaling [13]. In turn, Arf6 activates the MEK/ERK signaling pathway [14]. HCT116 cells display a gain of function mutation in KRas, resulting in a constitutively activated MEK/ERK signaling, whereas SW48 are wild type for KRas. Thus, we hypothesized, that in Pals1-depleted cells, Arf6 activity is increased, resulting in an enhanced transcription by activation of MEK/ERK in a KRas-mutant-sensitized background (HCT116 cells), whereas in KRas wild type cells, increased Arf6 activation is not sufficient to trigger a strong increase in its own transcription. Indeed, we found that inhibition of Arf6 in HCT116∆Pals1 cells results in a significant decrease of Arf6 expression, supporting our hypothesis (Supplementary Fig. 3f). However, although inhibition of ERK led to a decrease in Arf6 mRNA expression in Pals1-depleted HCT116 cells, the effect was not as strong as for Arf6 inhibition, indicating that differences in KRas-signaling might not the only differences between HCT116∆Pals1 and SW48∆Pals1.

### Pals1-mediated inhibition of cell migration is independent of its canonical binding partners

Pals1 is well characterized as a conserved regulator of apical-basal polarity and TJ formation by stabilizing the transmembrane protein Crb and linking it to the multiple PDZ-domain protein PATJ. However, re-introduction of Pals1 variants, which are either deficient in binding to Crb (deletion of the PDZ domain of Pals1, Pals1∆PDZ) or to PATJ (deletion of the N-terminal L27 domain, Pals1∆L27N) into HCT116∆Pals1 cells decreased Arf6 protein levels (Supplementary Fig. 3 g) and suppresses the cell migration phenotype similar to wild type Pals1 (Supplementary Fig. 3 h). These data suggest that Pals1 inhibits Arf6 to control Rac1-dependent cell migration independently of its canonical binding partners Crb and PATJ. This is in contrast to the reported role of Pals1 in TJ formation and apical-basal polarity: Downregulation of Pals1 has been shown to result in delayed formation of TJ [3], disturbed trafficking of E-Cad [15] and impaired contact inhibition in cultured mammalian cells [16, 17]. All these functions are assigned to Pals1 as part of the Crb complex, as deletion of Crb in mice or downregulation of Crb or PATJ in cell culture results in similar phenotypes [1, 2, 4].

In HCT116 cells, only a minor fraction of Pals1 is localized to the TJ and deletion of Pals1 enhances collective cell migration of HCT116 clusters (in scratch assays) but also renders isolated HCT116 cells more motile without any effects on cell-cell contacts or assembled TJ.

## Conclusion

Pals1 functions as an inhibitor of Arf6/Rac1-mediated cell migration and invasion in vitro. Thereby, Pals1 inhibits metastasis of colorectal cancer cells in vivo. Notably, the canonical binding partners of Pals1, Crb and PATJ are not involved in this process. Low Pals1 expression correlates with poor survival in patients suffering from colorectal cancer. Our study identifies Pals1 as new key regulator of colorectal cancer progression.

## Supplementary Information

**Additional file 1: Supplementary Fig. 1.** Pals1-deficient HCT116 cells do not exhibit defects in cell-cell contacts but increased motility. **a** Western blot analysis of the expression level of Pals1 in HCT116 and CRISPR/Cas9 generated HCT116ΔPals1 cell line. **b-d** Immunostainings of confluent HCT116 and HCT116ΔPals1 cells stained against Pals1 (green in **b** and **c**), PATJ (red in **b**), Claudin7 (magenta in **c**), ZO-1 (red in **c**) and E-Cad (green in **d**). The ratio of membranous versus cytosolic E-Cad was quantified (*N* = 50). **e** Western blot analysis of the protein expression of Pals1 and E-Cadherin in HCT116 and HCT116ΔPals1 cells. **f** Live-cell imaging of individual cell migration trajectories of HCT116 and HCT116ΔPals1 on basal membrane matrix coated surface over 5 h. **g** Quantification of the velocity of the single cell tracking experiments (*N* = 60)**. h** Quantification of the translocation of the single cell tracking experiments (*N* = 60)**. i** Proliferation of HCT116 and HCT116ΔPals1 was evaluated over 8 days using an automated cell counter (*N* = 3). **j** Staining of confluent HCT116wt and HCT116ΔPals1 for DAPI (blue) and TUNEL (red) in order to detect apoptosis. Quantification of TUNEL-positive cells gave a mean of 0.23 ± 0.13% for wt and 0.52 ± 0.18% for Pals1-deficient cells (*N* = 3). Scale bars are 20 μm. **Supplementary Fig. 2.** Knockout of Pals1 results in increased Arf6 but not Arf1 expression. **a** Quantification of active Cdc42 from pulldown assays (*N* = 3). **b** Western blot analysis of phosphorylated PAK1/2, which is induced by active Rac1. **c** Real time quantitative PCR analysis of the mRNA expression of Arf6 in HCT116 and HCT116ΔPals1 cells (*N* = 3). **d** Quantification of active Arf6 normalized against total Arf6 from pulldown assays (*N* = 3). **e** Immunostaining of migrating HCT116wt cells with anti Pals1 (green), anti Arf6 (red) antibodies and Phalloidin-staining (magenta) in order to visualize F-actin. Arrow indicates lamellipodium, arrowhead points at a cell-cell-contact. **f** Western blot and quantification of pulldown assays for active Arf1 using GST-GGA3 (*N* = 3). GST-GGA3 is visualized by CBB staining. **g** Western blot demonstrating efficient knockdown of Arf6 in Pals1-deficient HCT116 cells by two different shRNAs against Arf6. A scrambled shRNA (scr) was used as control. **h** Wound healing assay with HCT116∆Pals1 cells expressing scrambled shRNA or Arf6 shRNAs. (*N* > 8). Scale bar is 20 μm. **Supplementary Fig. 3.** Knockout of Pals1 in SW48 cells results in increased cell migration and enhanced Arf6/Rac1 activation. **a** Western blot demonstrating the efficient knockout of Pals1 in SW48 cells. **b** Quantification of wound healing assays of SW48 and SW48ΔPals1 cell lines (*N* = 3). **c** and **d** Western blot analysis and quantification of GST pulldown assays for active Rac1 (*N* = 3) (c) and active Arf6 (*N* = 4) (d). **e** Quantification of Arf6 expression in SW48 and SW48∆Pals1 cells normalized against β-Actin. **f** Expression of Arf6 mRNA in HCT116 and HCT116∆Pals1 cells incubated with either DMSO, the Arf6 inhibitor NAV2729 (10 μM) or the ERK-inhibitor ERK U0126 (10 μM) for 24 h (*N* = 3). **g** Western blot analysis of wild type and HCT116∆Pals1 cells transiently transfected with empty vector or indicated Pals1 constructs. **h** Quantification of wound healing assays of wild type HCT116 cell and HCT116ΔPals1 expressing either GFP alone (control) or indicated Pals1-GFP variants (*N* = 5). **Supplementary Movie 1 and 2.** Wild type HCT116 cells (**movie 1**) and Pals1-deficient HCT116 cells (**movie 2**) were seeded (20,000 cells / ml) 2 days before the experiment on a matrix containing laminin, fibronectin and collagen IV. The motility was visualized by recording pictures every 5 min over 5 h.

**Additional file 2: Methods.**

## Data Availability

Supplementary Figs. 1–2 and supplemental movies 1 and 2 can be accessed online.

## Abbreviations

Arf6: ADP-ribosylation factor 6
Crb: Crumbs
CRIB: Cdc42/Rac1 interactive binding
E-Cad: E-Cadherin
GEF: Guanine exchange factor
PAK: p21 activated kinase
Pals1: Protein associated with Lin-7 One
PATJ: Pals1-associated TJ protein
TJ: tight junction
