# Supplementary figures and images for: Pals1 prevents Rac1-dependent colorectal cancer cell metastasis by inhibiting Arf6
Source: Mol Cancer. 2021 May 4;20:74. doi: 10.1186/s12943-021-01354-2 (PMC8094600; doi:10.1186/s12943-021-01354-2)

**a**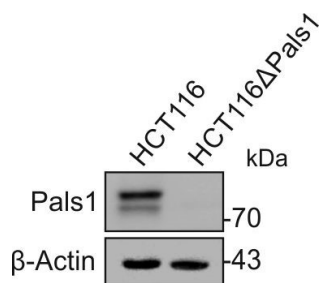**b**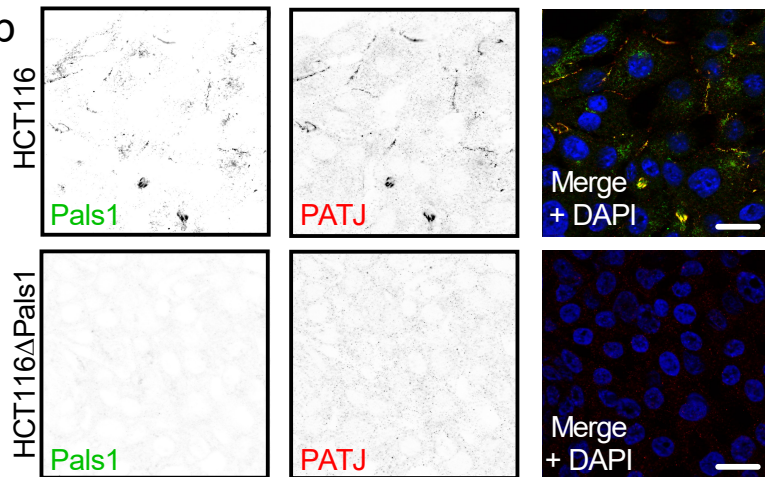**c**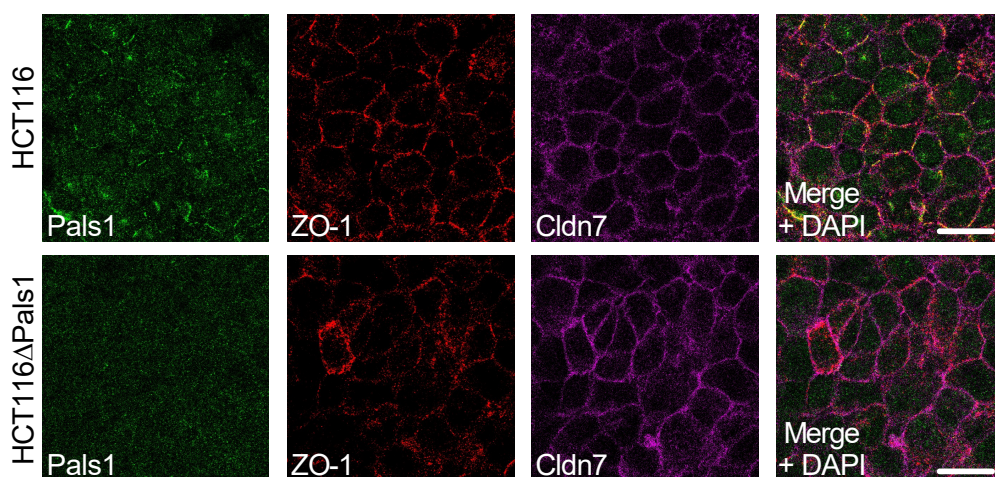**d**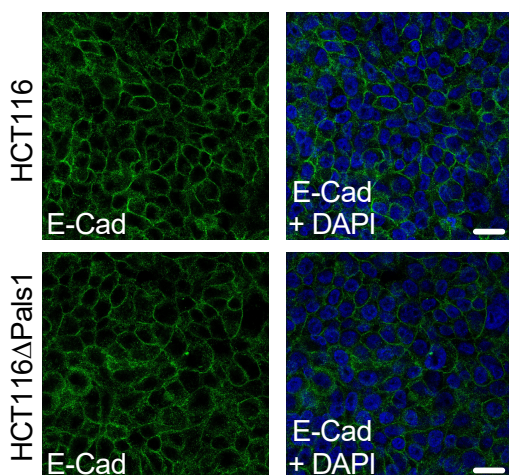

Ratio membranous/cytosolic E-Cad

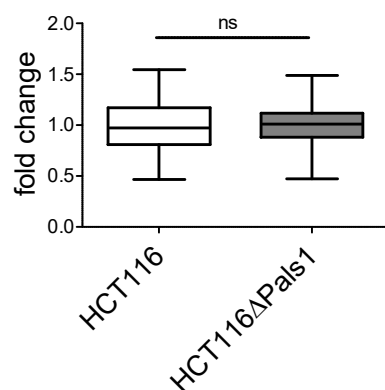**e**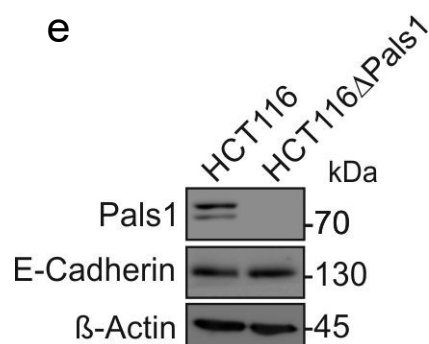**f**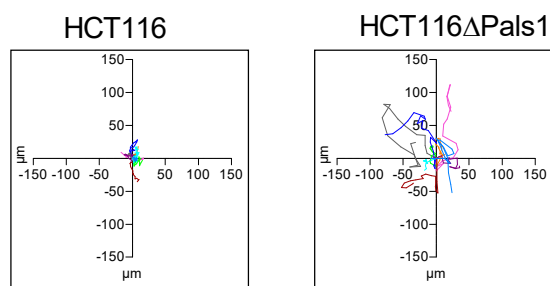**g**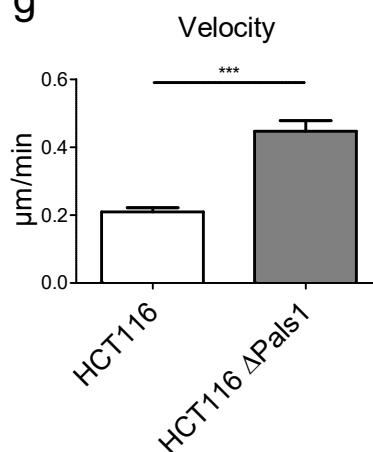**h**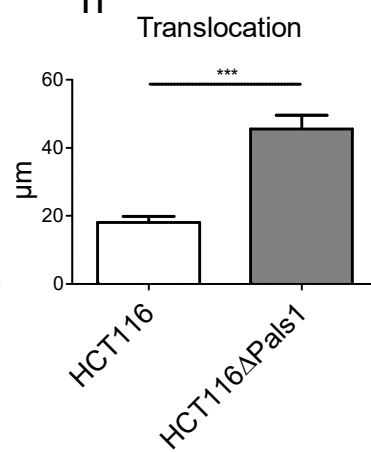**i**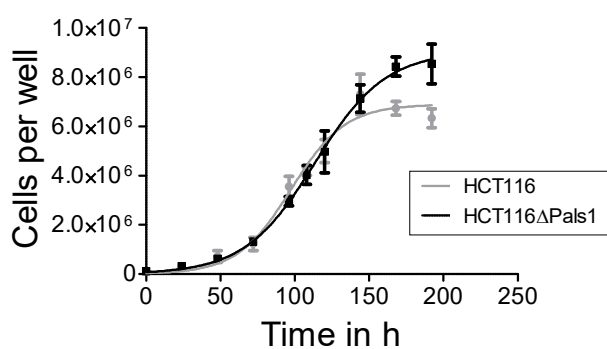**j**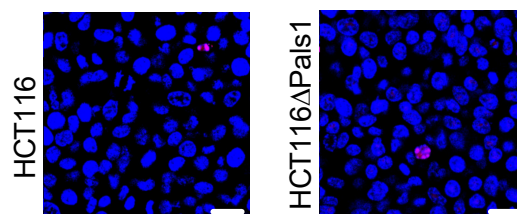

Supplement: Supplementary file 1 — Additional file 1: Supplementary Fig. 1. Pals1-deficient HCT116 cells do not exhibit defects in cell-cell contacts but increased motility. a Western blot analysis of the expression level of Pals1 in HCT116 and CRISPR/Cas9 generated HCT116ΔPals1 cell line. b-d Immunostainings of confluent HCT116 and HCT116ΔPals1 cells stained against Pals1 (green in b and c), PATJ (red in b), Claudin7 (magenta in c), ZO-1 (red in c) and E-Cad (green in d). The ratio of membranous versus cytosolic E-Cad was quantified (N = 50). e Western blot analysis of the protein expression of Pals1 and E-Cadherin in HCT116 and HCT116ΔPals1 cells. f Live-cell imaging of individual cell migration trajectories of HCT116 and HCT116ΔPals1 on basal membrane matrix coated surface over 5 h. g Quantification of the velocity of the single cell tracking experiments (N = 60). h Quantification of the translocation of the single cell tracking experiments (N = 60). i Proliferation of HCT116 and HCT116ΔPals1 was evaluated over 8 days using an automated cell counter (N = 3). j Staining of confluent HCT116wt and HCT116ΔPals1 for DAPI (blue) and TUNEL (red) in order to detect apoptosis. Quantification of TUNEL-positive cells gave a mean of 0.23 ± 0.13% for wt and 0.52 ± 0.18% for Pals1-deficient cells (N = 3). Scale bars are 20 μm. Supplementary Fig. 2. Knockout of Pals1 results in increased Arf6 but not Arf1 expression. a Quantification of active Cdc42 from pulldown assays (N = 3). b Western blot analysis of phosphorylated PAK1/2, which is induced by active Rac1. c Real time quantitative PCR analysis of the mRNA expression of Arf6 in HCT116 and HCT116ΔPals1 cells (N = 3). d Quantification of active Arf6 normalized against total Arf6 from pulldown assays (N = 3). e Immunostaining of migrating HCT116wt cells with anti Pals1 (green), anti Arf6 (red) antibodies and Phalloidin-staining (magenta) in order to visualize F-actin. Arrow indicates lamellipodium, arrowhead points at a cell-cell-contact. f Western blot and quan [file 12943_2021_1354_MOESM1_ESM.zip › Figure S1.pdf]

a

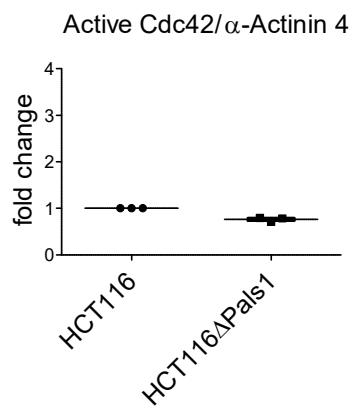

b

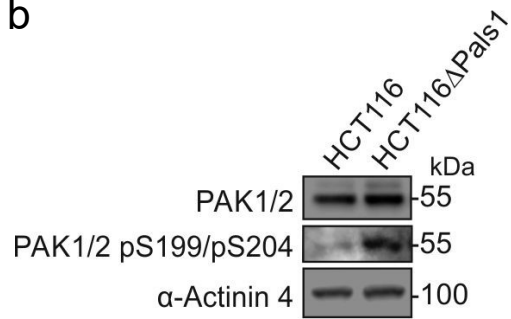

c

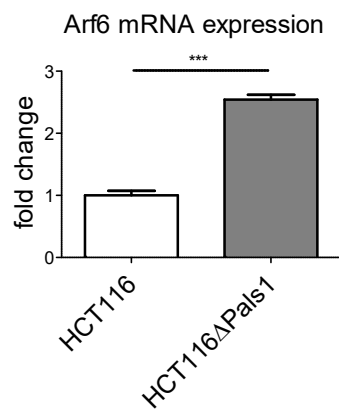

d

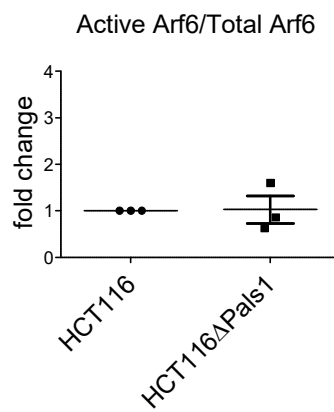

e

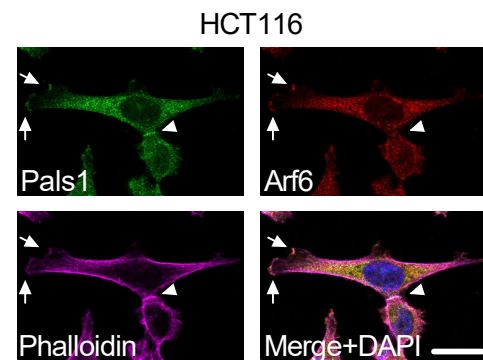

f

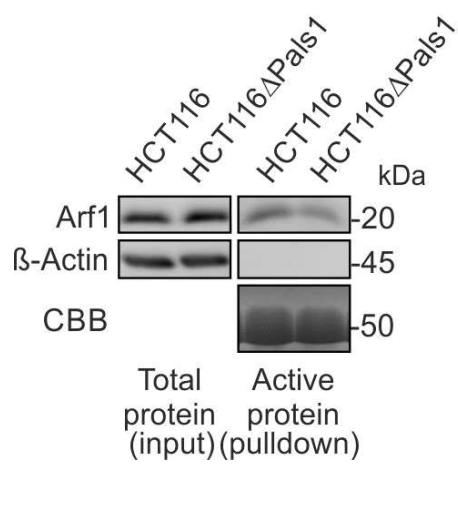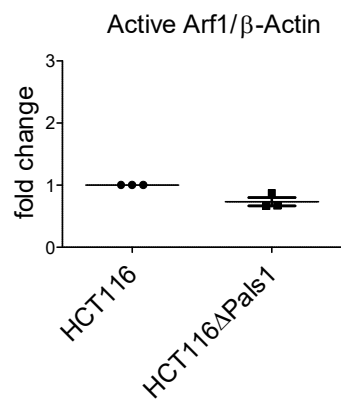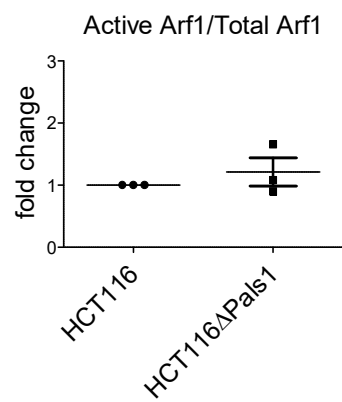

g

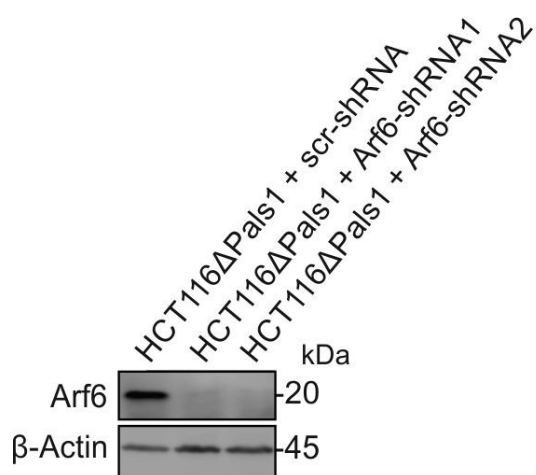

h

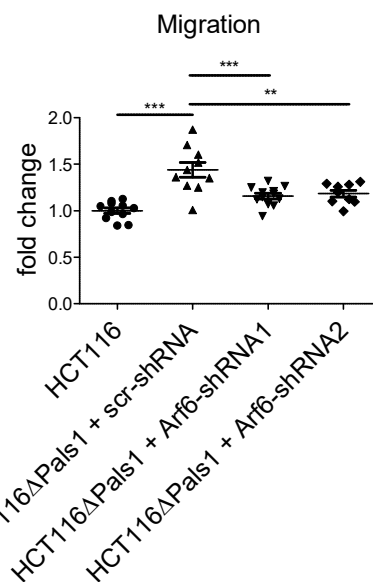

Supplement: Supplementary file 1 — Additional file 1: Supplementary Fig. 1. Pals1-deficient HCT116 cells do not exhibit defects in cell-cell contacts but increased motility. a Western blot analysis of the expression level of Pals1 in HCT116 and CRISPR/Cas9 generated HCT116ΔPals1 cell line. b-d Immunostainings of confluent HCT116 and HCT116ΔPals1 cells stained against Pals1 (green in b and c), PATJ (red in b), Claudin7 (magenta in c), ZO-1 (red in c) and E-Cad (green in d). The ratio of membranous versus cytosolic E-Cad was quantified (N = 50). e Western blot analysis of the protein expression of Pals1 and E-Cadherin in HCT116 and HCT116ΔPals1 cells. f Live-cell imaging of individual cell migration trajectories of HCT116 and HCT116ΔPals1 on basal membrane matrix coated surface over 5 h. g Quantification of the velocity of the single cell tracking experiments (N = 60). h Quantification of the translocation of the single cell tracking experiments (N = 60). i Proliferation of HCT116 and HCT116ΔPals1 was evaluated over 8 days using an automated cell counter (N = 3). j Staining of confluent HCT116wt and HCT116ΔPals1 for DAPI (blue) and TUNEL (red) in order to detect apoptosis. Quantification of TUNEL-positive cells gave a mean of 0.23 ± 0.13% for wt and 0.52 ± 0.18% for Pals1-deficient cells (N = 3). Scale bars are 20 μm. Supplementary Fig. 2. Knockout of Pals1 results in increased Arf6 but not Arf1 expression. a Quantification of active Cdc42 from pulldown assays (N = 3). b Western blot analysis of phosphorylated PAK1/2, which is induced by active Rac1. c Real time quantitative PCR analysis of the mRNA expression of Arf6 in HCT116 and HCT116ΔPals1 cells (N = 3). d Quantification of active Arf6 normalized against total Arf6 from pulldown assays (N = 3). e Immunostaining of migrating HCT116wt cells with anti Pals1 (green), anti Arf6 (red) antibodies and Phalloidin-staining (magenta) in order to visualize F-actin. Arrow indicates lamellipodium, arrowhead points at a cell-cell-contact. f Western blot and quan [file 12943_2021_1354_MOESM1_ESM.zip › Figure S2.pdf]

a

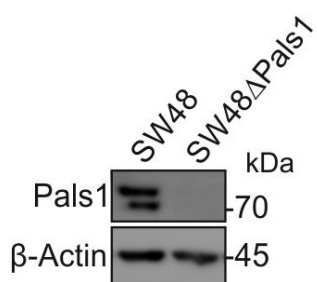

b

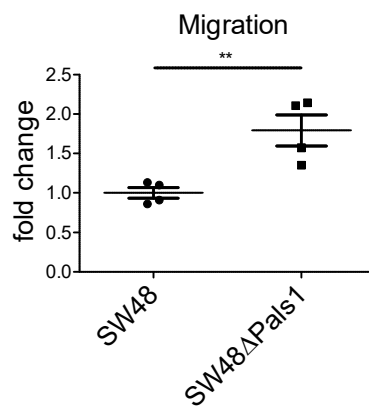

c

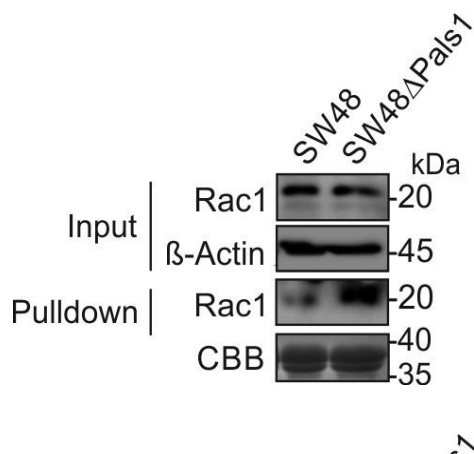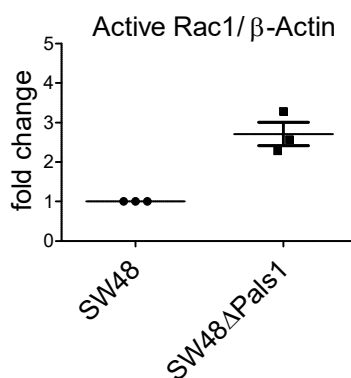

d

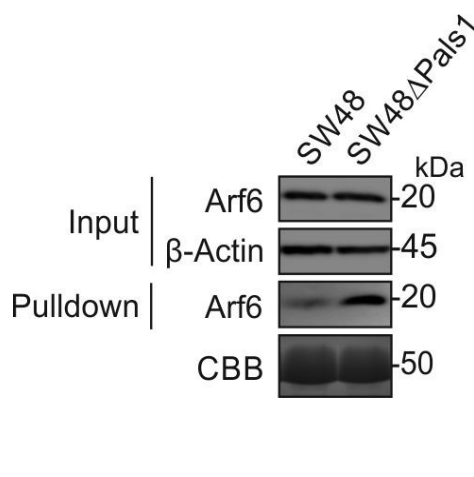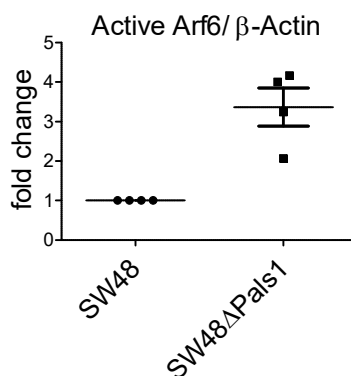

e

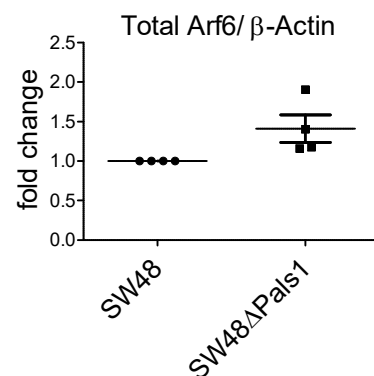

f

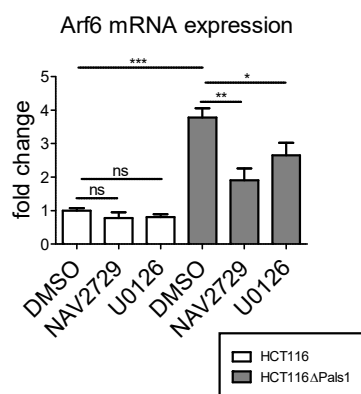

g

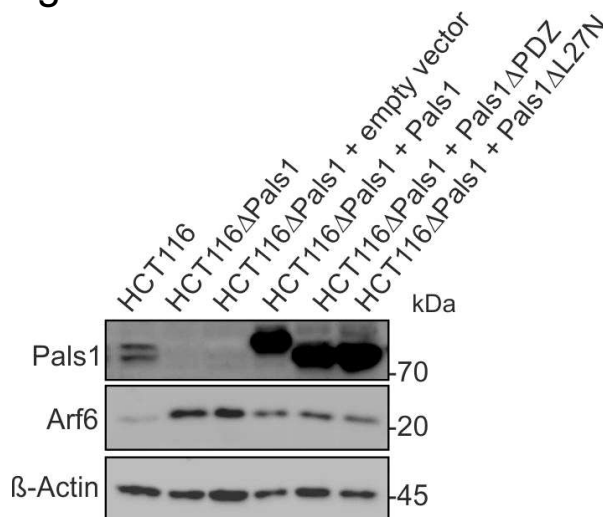

h

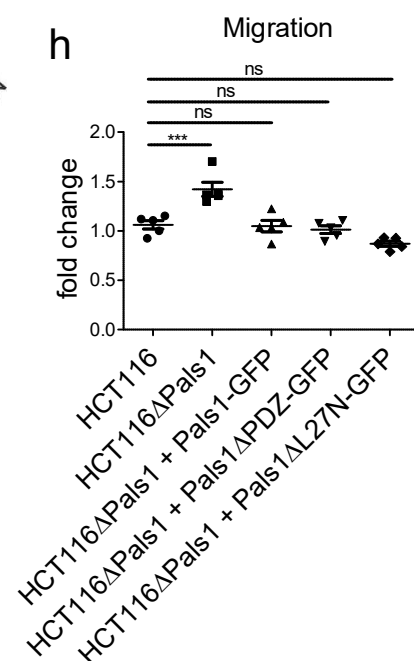

Supplement: Supplementary file 1 — Additional file 1: Supplementary Fig. 1. Pals1-deficient HCT116 cells do not exhibit defects in cell-cell contacts but increased motility. a Western blot analysis of the expression level of Pals1 in HCT116 and CRISPR/Cas9 generated HCT116ΔPals1 cell line. b-d Immunostainings of confluent HCT116 and HCT116ΔPals1 cells stained against Pals1 (green in b and c), PATJ (red in b), Claudin7 (magenta in c), ZO-1 (red in c) and E-Cad (green in d). The ratio of membranous versus cytosolic E-Cad was quantified (N = 50). e Western blot analysis of the protein expression of Pals1 and E-Cadherin in HCT116 and HCT116ΔPals1 cells. f Live-cell imaging of individual cell migration trajectories of HCT116 and HCT116ΔPals1 on basal membrane matrix coated surface over 5 h. g Quantification of the velocity of the single cell tracking experiments (N = 60). h Quantification of the translocation of the single cell tracking experiments (N = 60). i Proliferation of HCT116 and HCT116ΔPals1 was evaluated over 8 days using an automated cell counter (N = 3). j Staining of confluent HCT116wt and HCT116ΔPals1 for DAPI (blue) and TUNEL (red) in order to detect apoptosis. Quantification of TUNEL-positive cells gave a mean of 0.23 ± 0.13% for wt and 0.52 ± 0.18% for Pals1-deficient cells (N = 3). Scale bars are 20 μm. Supplementary Fig. 2. Knockout of Pals1 results in increased Arf6 but not Arf1 expression. a Quantification of active Cdc42 from pulldown assays (N = 3). b Western blot analysis of phosphorylated PAK1/2, which is induced by active Rac1. c Real time quantitative PCR analysis of the mRNA expression of Arf6 in HCT116 and HCT116ΔPals1 cells (N = 3). d Quantification of active Arf6 normalized against total Arf6 from pulldown assays (N = 3). e Immunostaining of migrating HCT116wt cells with anti Pals1 (green), anti Arf6 (red) antibodies and Phalloidin-staining (magenta) in order to visualize F-actin. Arrow indicates lamellipodium, arrowhead points at a cell-cell-contact. f Western blot and quan [file 12943_2021_1354_MOESM1_ESM.zip › Figure S3.pdf]
