# Supplementary material for: Pals1 prevents Rac1-dependent colorectal cancer cell metastasis by inhibiting Arf6
Source: Mol Cancer. 2021 May 4;20:74. doi: 10.1186/s12943-021-01354-2 (PMC8094600; doi:10.1186/s12943-021-01354-2)
Supplement: Supplementary file 2 — Additional file 2: Methods. [file 12943_2021_1354_MOESM2_ESM.pdf]

Methods

Cell culture

HCT116/HCT116ΔPals1 and were cultured in DMEM high glucose medium (4.5 g/L, Sigma-Aldrich), SW48/SW48ΔPals1 cells in RPMI medium (Sigma-Aldrich), both supplemented with 10% FCS and 1% antibiotics (streptomycin/penicillin). All cell lines were cultivated at 37°C under 5% CO_2 atmosphere and passaged every 3-4 days. Transfections were performed with Lipofectamin™ 2000 (Thermo Fisher Scientific) or Metafectene® (Biontex) according to the manufacturer's introductions.

To establish stable Pals1 HCT116 and SW48 knockout cells lines by using CRISPR/Cas9, cells were transfected with one of the following guides together with Cas9 from px459: GCCCTGGAGATTTGGGCACC and ATTAGCCGGATAGTAAAAGG. Non transfected cells were eliminated with 36 h puromycin selection and subsequently single-cell-clones were generated and analyzed for efficient knockout by western blot (Supplementary Fig. S1a and S3a) and sequencing. For HCT116ΔPals1, we found a deletion (ATTCGGCAAC), which results in a frame shift and a premature stop codon at aa 66. In SW48ΔPals1, a single bp was deleted (underlined): ATAGTAAA<u>A</u>GGGGGTGC, resulting in a frameshift and a premature stop codon at aa 302. No Pals1 band is detectable in western blots of both cell lines using an antibody recognizing the N-terminus of the protein, indicating that the truncated proteins are instable. Stable rescue cell lines were established by using the retroviral pQXCIP system. In order to downregulate Arf6 expression in HCT116ΔPals1 cells, the following shRNA sequences were used in pLKO-Puro: Arf6 shRNA#1 GGGGAAGGTGCTATCCAAAAT, Arf6 shRNA#2 GGTTAACCTCTAACTACAAAT, scrambled (control) shRNA GCGCGATAGCGCTAATAATTT. The following inhibitors were used in this study: Rac1 inhibitor EHT1864 (5µM, Santa Cruz), Arf6 inhibitor NAV2729 (10µM, Tocris Bioscience) and ERK inhibitor U0126 (10µM, SIGMA-Aldrich).

Immunofluorescence analysis

Cells were grown on coverslips and fixed with 4% PFA in phosphate buffer pH 7.4 for 7 min or with methanol at -20°C for 10 min. After washing three times with PBS cells were incubated for 1 h with PBS + 2.5% horse serum and 0.05% Saponin or 0.1% Triton X-100. Subsequently, primary antibodies were diluted in the same solution for 2 h at RT or overnight at 4°C. After washing three times with PBST/PBSS the coverslips were incubated with the secondary antibody (diluted 1:1000 in PBSS+HS/PBST+HS), DAPI (1:1000, Invitrogen Life Technologies) and Phalloidin (1:1000, Santa Cruz #363796) for 1 h. Finally, coverslips were washed with PBS and mounted in mowiol. The following primary antibodies were used: mouse anti Pals1 (1:100, Santa Cruz #365411), rabbit anti Pals1 (1:1000, Proteintech #17710-1AP), mouse anti Rac (1:100, Santa Cruz #514583), rabbit anti Arf6 (1:100, Proteintech 20225-1-AP), rabbit anti PATJ (1:100, raised in this study), goat anti Claudin7 (1:100, Santa Cruz #17670), rabbit anti ZO-1 (1:100, Cell Signaling #13663), rabbit anti E-Cadherin (1:100, Cell Signaling #3195), mouse anti E-Cadherin (1:100, Sanza Cruz # 21791). In order to detect apoptosis in HCT116 and HCT116ΔPals1 cells, the in situ cell death detection kit-TMRed (SIGMA-Aldrich, #12156792910) was used.

Immunohistochemistry

Tissue samples of the liver and lung of HCT116wt and HCT116ΔPals1 cells transplanted NSG mice were fixed in 4% formalin and embedded in paraffin. A Tissue Micro Array (TMA) of paraffin-embedded healthy colon samples and high-grade colorectal tumors was assembled from patient material collected at the University Medical Center of Regensburg from 1997 to 2015. Paraffin sections were deparaffinized for 30 min at 72°C, washed two times for 7 min in Xylol and subsequently re-watered in a descending sequence of ethanol/water mixture. Prior to staining, sections were subjected for 5 min to heat-induced epitope-retrieval (HIER) using 1mM

Tris-EDTA-buffer (pH 8.5) at 120°C. Sections were blocked in peroxidase-blocking solution (Dako, #S2023) for 5 min at RT and washed 5 min with wash buffer (Dako, #S3006) prior to incubation with primary antibody diluted in antibody diluent (Dako, #S2022) for 30 min. Primary antibodies were as follows: mouse anti-E-Cadherin (1:100, NCH-38, DAKO), mouse anti-Pals1 (1:10, Santa Cruz #sc-365411). Stainings were developed after washing using DAB/chromogen solution (Dako, #K5007). To visualize cellular structures, stained sections were counterstained with hematoxylin (Merck, #10517505000) for 1 min and dehydrated in ethanol/xylol before embedding.

Real-time PCR analysis

RNA was isolated using the GenElute™ Mammalian Total RNA Miniprep Kit (Sigma-Aldrich). 2 µg total RNA was used for cDNA synthesis (SuperScript-III First-Strand Synthesis SuperMix (LifeTechnologies)). Real time PCR was carried out in the CFX384 Realtime Detection System (Biorad) using FastStart Universal SYBR Green Master (Thermo Fisher). Relative gene expression values were analysed with the $2^{-\Delta\Delta Ct}$ method with GAPDH as housekeeping gene. The following primers were used: hARF6-F: 5’-TCAAGTTGTGCGGTCGGTGA-3’, hArf6-R: 5’-GCGAAACCGAAACGAAGCCT-3’, hGAPDH-F: 5’-CAAGCTCATTTCCTGGTATGAC-3’ and hGAPDH-R: 5’-GTGTGGTGGGGGACTGAGTGTGG-3’.

Cell lysates and western blot

Cell lysates were made with Laemmli buffer (western blot) or with TNT-buffer (pulldown experiments, 150 mM NaCl, 50 mM Tris, 1% Triton, pH 7.4 + protease inhibitor cocktail). SDS-PAGE and western blotting was performed according standard procedures. The following primary antibodies were used: mouse anti Pals1 (1:500, Santa Cruz #365411), rabbit anti Pals1 (1:1000, Proteintech 17710-1AP), mouse anti ß-Actin (1:500, Santa Cruz #47778), mouse anti

Cdc42 (1:500, Santa Cruz #8401), mouse anti Rac (1:500, Santa Cruz #514583), rabbit anti α-Actinin 4 (1:1000, Cell Signaling #6487), mouse anti PAK1/2 (1:500, Santa Cruz #166174), rabbit anti Pak1/2 pS199/pS204 (1:1000, Cell Signaling #2605S), rabbit anti Arf1 (1:1000, Proteintech #10790-1AP), mouse anti Arf6 (1:500, Santa Cruz #7971), rabbit anti E-Cadherin (1:1000, Santa Cruz #7870).

Recombinant protein expression and GST pulldown

Recombinant proteins were expressed using *E.coli* BL21* bacteria. Proteins were harvested after lysis of cells with TNT-buffer (50 mM TRIS-HCl pH 7.4, 150mM NaCl, 1% Triton-X100) and sonification using GST beads (for GST-Arf6, GST-GGA3, GST-PBD, Macherey-Nagel). TNT cell lysates (400 µl) from a confluent 10 cm plate were used for the GTPase activity pulldowns by incubation of the recombinant proteins with lysates for 2 h at 4°C. After three washing steps, beads were boiled in Laemmli buffer and proteins submitted to SDS-PAGE and western blotting.

Wound healing assay

Cells in 6-wells were separated by culture-Inserts (2 well, Ibidi) until confluency. Assays were started with the removal of the insert and pictures taken by a ZEISS Observer Z1. Data were analysed using ImageJ.

Transwell matrigel invasion assay

Thincert cell culture inserts (8 µm, Greiner Bio-One) were coated with Cultrex® basement membrane extract (BME, final concentration 2 mg/ml, diluted in DMEM). After incubation at 37°C for 2 h 1.5 ml of DMEM with 10 % FCS were added to the lower compartment. 100 000 cells in serum free medium were added to the upper compartment and incubated at 37°C. Subsequently remaining cells in the upper compartment were removed and cells in the lower

compartment were stained with crystal violet after PFA fixation. For quantification, cells were removed from the membrane with methanol and OD570 was measured the microplate readers infinite® M200 (Tecan Trading AG).

Single-cell migration assay

Two days before the experiment HCT116 and HCT116Δcell (20 000 cells/ml) were seeded on a T25 culture flask (Corning) precoated with laminin (1 mg/ml, Sigma-Aldrich), fibronectin (1 mg/ml, Roche) and collagen IV (0.9 mg/ml, VWR). Single cell migration was performed in temperature-controlled cambers (37°C) using a Zeiss Axiovert D40 microscope. Migration was recorded over 5 h in 5 min intervals and analyzed with Amira 2019.2 and Matlab software. For each independent experiment (N) 10 single cells (n) were analyzed.

Xenotransplantation assay

NOD-*scid* $IL2R\gamma^{null}$ (NSG) immunodeficient mice were obtained from Jackson Laboratories and housed in a specialized pathogen-free facility at the University of Regensburg. 2-3 days old offspring of these mice were irradiated (1 Gy) and 3 hours later injected subcutaneously with 100 000 HCT116wt and HCT116ΔPals1 cells. The tumor growth was monitored twice a week and the tumor size was measured. After 5 weeks the animals were sacrificed, and the number of metastases were estimated in lungs and livers by immunohistochemistry using mouse anti pan-cytokeratin (A1/A3 antibody mix 1:100) as marker. The animal work was approved by the local veterinary authorities of the district government based on the European guidelines and national regulations of the German Animal Protection Act (approval no. DMS-2532-2-34).

Statistical analysis

All data is presented as mean±SEM of at least three independent experiments. Statistical significance was evaluated with unpaired t-test or ANOVA using GraphPad Prism: ns > 0.05, * $p < 0.05$, ** $p < 0.01$, *** $p < 0.001$.
